# Case Report WIN-MTB-2024001 – WIN International Molecular Tumor Board: A 55-year-old male with glioblastoma exhibiting amplifications in MDM2 and EGFR

**DOI:** 10.18632/oncotarget.28920

**Published:** 2026-10-16

**Authors:** Wafik S. El-Deiry, Sadakatsu Ikeda, Shai Magidi, Catherine Bresson, Hikmat Abdel-Razeq, Anthony Joshua, Alberto Hernando-Calvo, Arjun Oberoi, Zhihuang Hu, Tobias Meissner, Rachel Elsey, Amal Al-Omari, Fanny Wunder, Razelle Kurzrock

**Affiliations:** ^1^Worldwide Innovative Network (WIN) Association - WIN Consortium, Chevilly-Larue 94550, France; ^2^Legorreta Cancer Center at Brown University, Providence, RI 02912, USA; ^3^Institute of Science Tokyo Hospital, Tokyo 113-8510, Japan; ^4^Fudan University Shanghai Cancer Center, Shanghai 200032, China; ^5^King Hussein Cancer Center, Amman 11941, Jordan; ^6^The Kinghorn Cancer Centre, St Vincent's Hospital, Darlinghurst, NSW 2010, Australia; ^7^Vall d'Hebron Hospital Campus and Institute of Oncology (VHIO), Barcelona 08035, Spain; ^8^Avera Cancer Institute, Sioux Falls, SD 57105, USA; ^9^Medical College of Wisconsin, Milwaukee, WI 53226, USA; ^*^These authors contributed equally to this work

**Keywords:** cancer, precision oncology, molecular tumor board, glioblastoma

## Abstract

The WIN Consortium International Molecular Tumor Board brings together precision oncology experts from 18 organizations in 13 countries across four continents and provides discussion for patients with cancer across the world. This case report details the medical journey of a 55-year-old man diagnosed with high-grade glioblastoma (GBM), characterized by amplifications of *MDM2* and *EGFR*, alongside other critical genomic alterations, including a *CDKN2A* frameshift mutation. Despite the aggressive nature of GBM, the patient was in remission following a combination of radiotherapy and chemotherapy, surgery and participation in a phase II clinical trial (PI3K/mTOR inhibitor) combined with ketogenic diet and metformin. However, the patient was also considering personalized neoantigen vaccine therapy targeting tumor-specific mutated peptides as part of his treatment strategy.

This case underscores the immense complexity of treating GBM. Next generation sequencing showed *MDM2* amplification, which hinders the tumor-suppressive functions of p53, and *EGFR* amplification, which promotes oncogenic signaling. The experts highlighted various therapeutic options discussed in the event of relapse: *EGFR* inhibitors, various *MDM2* inhibitors, and personalized combination therapies targeting both *EGFR* and *CDK4/6*. Additionally, the Molecular Tumor Board discussed the reported risk of resistance or hyper-progression with immune checkpoint blockade in the presence of *EGFR* and *MDM2* alterations. Innovative approaches such as combining *MDM2* and *EGFR* inhibitors with immunotherapy, as well as the use of teserpaturev, a genetically engineered herpes simplex virus were addressed. This case emphasizes the importance of personalized treatment strategies for GBM and the need for ongoing clinical research to explore novel therapeutic options.

## INTRODUCTION

Glioblastoma (GBM) remains one of the most aggressive and lethal primary brain tumors in adults [1–3]. In the current WHO classification, glioblastoma is defined as a diffuse astrocytic glioma, IDH-wildtype, CNS WHO grade IV [4]. Characterized by rapid tumor growth and diffuse infiltration, GBM presents significant challenges in terms of treatment and prognosis [3, 5–8]. Although there have been advancements in multimodal treatments including surgical resection, radiotherapy, and chemotherapy, the median survival for GBM patients remains approximately 15 months and the five-year survival rate is around 5–10% [9, 10].

The molecular heterogeneity of GBM contributes to its resistance to therapy and underscores the importance of studying specific genetic alterations that drive tumor progression [11, 12]. *MDM2*, frequently amplified in high-grade gliomas, acts as a negative regulator of p53, a key tumor suppressor that controls the cell cycle [13]. By binding to p53 and promoting its degradation, *MDM2* impairs p53’s tumor-suppressive functions, driving tumorigenesis, uncontrolled cell proliferation, resistance to apoptosis, and enhanced survival of glioma cells [14, 15]. Elevated *MDM2* expression correlates with tumor grade and poor prognosis [16]. Given its pivotal role in suppressing p53, *MDM2* has emerged as a therapeutic target in high-grade gliomas. Inhibiting *MDM2* is being explored as a strategy to reactivate p53, restoring its potent tumor-suppressive functions and potentially reversing tumor progression [15]. Also, *MDM2* overexpression is associated with chemoresistance, particularly to drugs that rely on p53-mediated apoptosis [17]. This is especially problematic in GBM, which is commonly treated with chemotherapy agents like temozolomide. Therefore, *MDM2* inhibition has been proposed as a potential strategy to sensitize glioma cells to chemotherapy by reactivating p53 and enhancing the response to agents like temozolomide. Although preclinical models have shown encouraging results with *MDM2* inhibitors, clinical applications are still under investigation [18].

Beyond its regulation of p53, *MDM2* is also involved in other pathways critical to glioma progression, including cell cycle regulation, DNA repair, and cell survival [14]. The *MDM2*-p53 pathway interacts with multiple other pathways in gliomas, and alterations in these regulatory proteins collectively contribute to tumor progression and resistance to therapy [19]. *MDM2* interacts with various proteins, including retinoblastoma (RB) and alternate reading frame (p14ARF), and is influenced by cellular signaling pathways such as epidermal growth factor receptor (EGFR) signaling, which is often dysregulated in GBM [14, 20–23]. Amplification of *EGFR* drives oncogenic signaling pathways that promote proliferation, invasion, angiogenesis, and resistance to therapy [20]. Both *MDM2* and *EGFR* alterations are associated with aggressive tumor behavior and worse clinical outcomes [14, 21, 24]. Studies have shown that *MDM2* amplification occurs in approximately 10–14% of GBMs, while *EGFR* amplification is observed in approximately 50% of cases [25–27].

*CDKN2A* is another important tumor suppressor gene that regulates the cell cycle, preventing uncontrolled cell proliferation by producing two different tumor-suppressor proteins – (i) p16INKa, which blocks CDK4/6 and helps to keep the RB pathway off and prevent cells from entering the S-phase too easily, and (ii) p14ARF which restrains MDM2 and helps keep p53 active when the cell is stressed/damaged [28–30]. Molecular alterations in *CDKN2A*, including loss of heterozygosity and deletions, are commonly observed in GBM, where they drive tumor progression and correlate with poor prognosis [31, 32].

This paper presents a case of a 55-year-old male patient diagnosed with high-grade GBM, exhibiting amplifications of both *MDM2* and *EGFR*, along with other molecular aberrations such as *CDKN2A* frameshift mutation and loss of heterozygosity. We provide detailed account of the patient’s clinical history, molecular profiling and treatment course.

An international Molecular Tumor Board (MTB), composed of expert physicians and scientists specializing in personalized cancer medicine from 18 centers across 13 countries as shown in Figure 1, convened by the Worldwide Innovative Network (WIN) Association - WIN Consortium, discussed the case. While the patient was in remission at the time of the discussion, the MTB considered potential therapeutic options should the disease progress. This report builds on prior published WIN Consortium’s International MTB case reporting [33].

**Figure 1:**
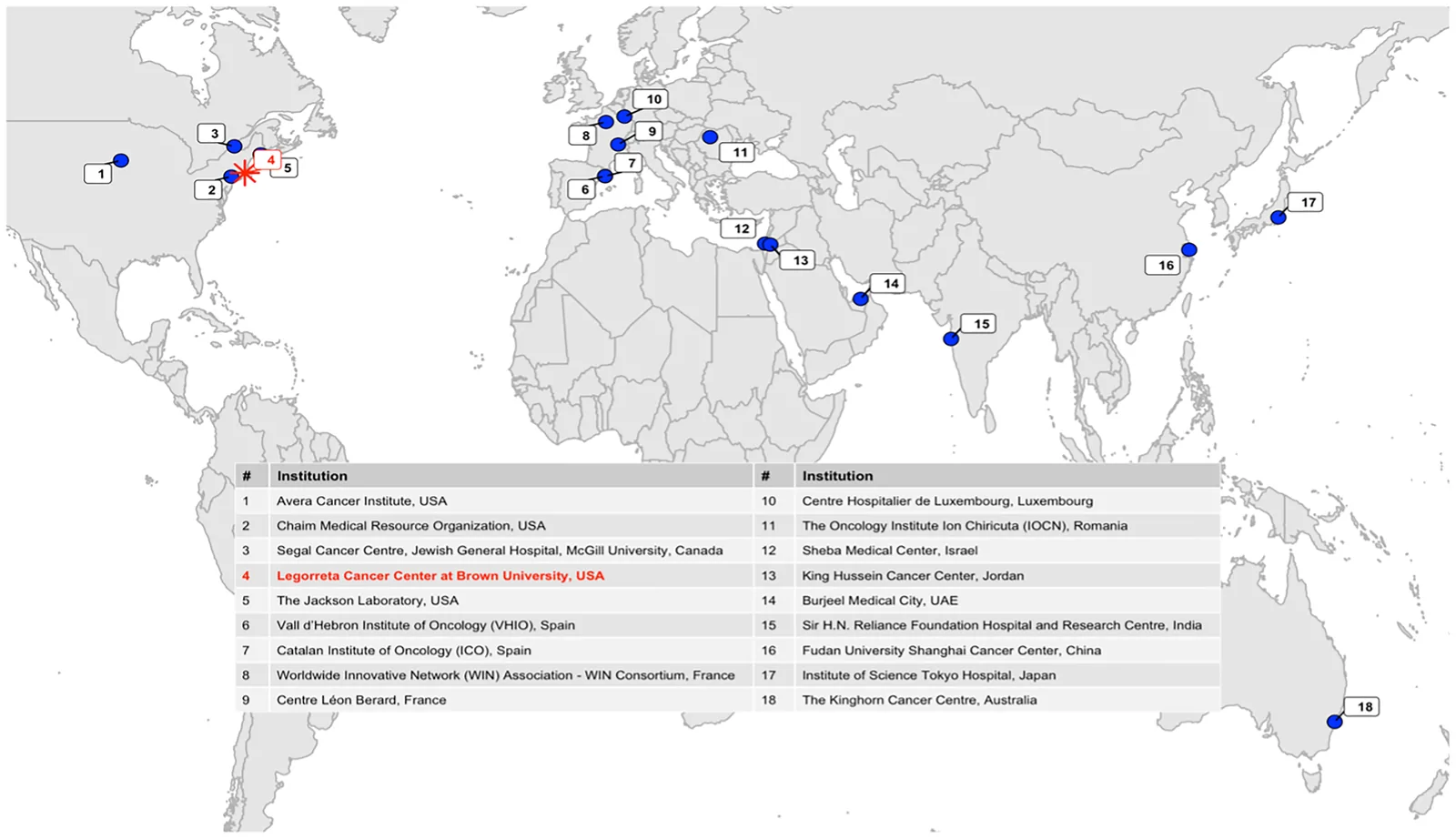
WIN Consortium International Molecular Tumor Board participants. The figure highlights 18 institutions participating from 13 countries worldwide (blue dots), with the patient discussed in the MTB originating from USA (red Asterisk).

## CASE REPORT DESCRIPTION

The patient, a 55-year-old man, was diagnosed with high-grade GBM in June 2023. He was generally healthy with a medical history of hypertension and hyperlipidemia. He had a 10-pack year tobacco history but had quit 20 years ago.

The patient’s medical journey began in February 2023, when he experienced dizziness while driving and presented to the emergency room, where he subsequently had a generalized tonic-colonic (GTC) seizure. Following this episode, he suffered from impaired speech. MRI and CT scans of the brain revealed a small mass in the left temporal area, as shown in Figure 2A. However, no immediate action was necessary at that time, and he was discharged with a 10-day course of anti-epileptic medication.

**Figure 2:**
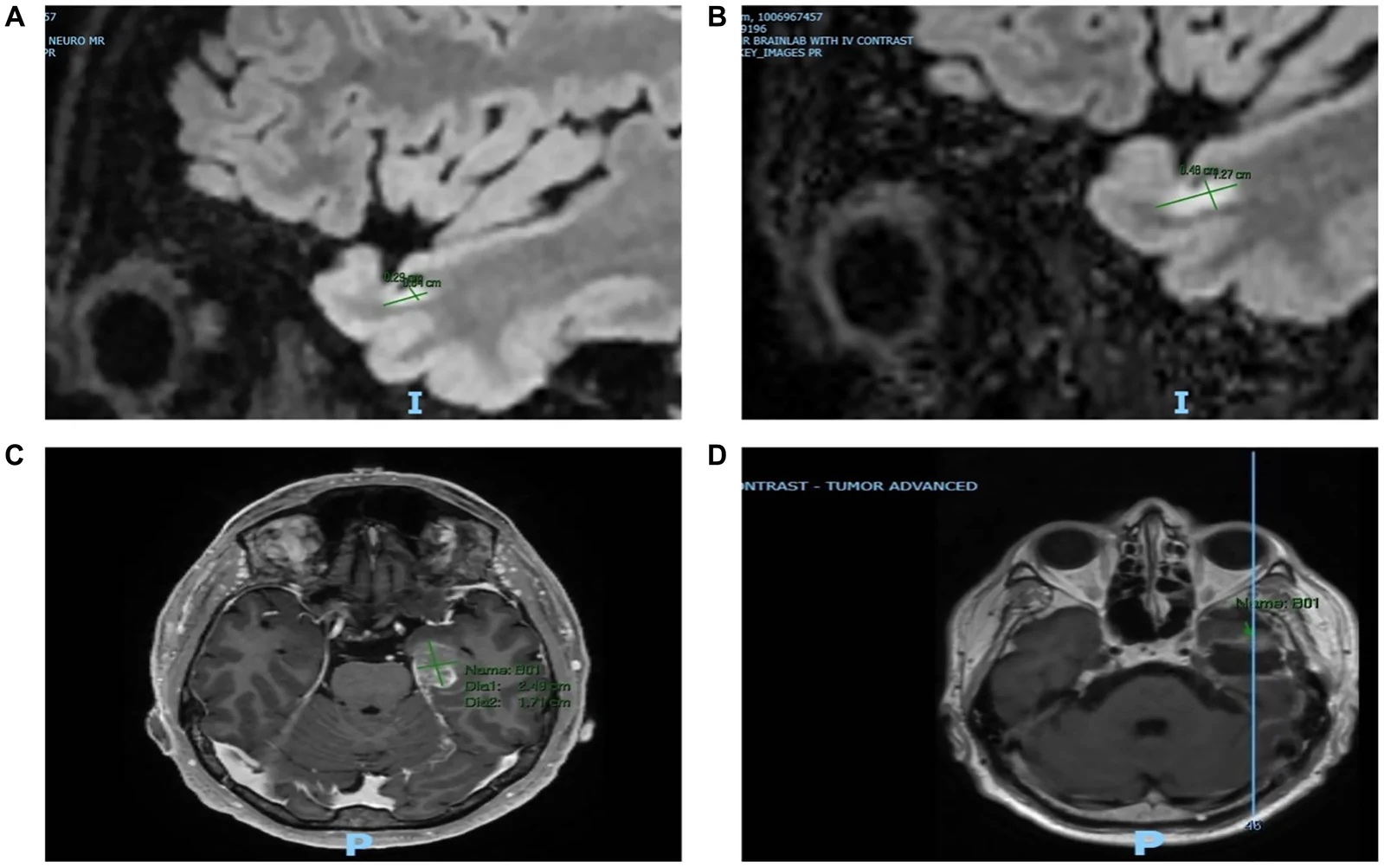
MRI brain scan images. (**A**) February 2023 - A mass measuring 0.29 × 0.04 cm in the left temporal lobe. (**B**) June 2023 - The mass has enlarged to 1.27 × 0.48 cm in the left temporal lobe. (**C**) June 2023 – A hyperintense mass in the left medial temporal lobe, measuring 2.49 × 1.71 cm, infiltrating the hippocampus, amygdala, and anterior temporal lobe. (**D**) June 2023, post-surgery – Nodular enhancement on the resection site, with a small residual mass not excluded.

By June 2023, the patient had another episode of GTC seizure, this time with a loss of consciousness. A follow-up MRI revealed that the left temporal lobe mass had grown, as shown in Figure 2B, 2C. The patient underwent neurosurgical procedure consisting of anterior temporal lobectomy and amygdalohippocampectomy, as outlined in Figure 3. The MRI scan post-surgery is shown in Figure 2D.

**Figure 3:**
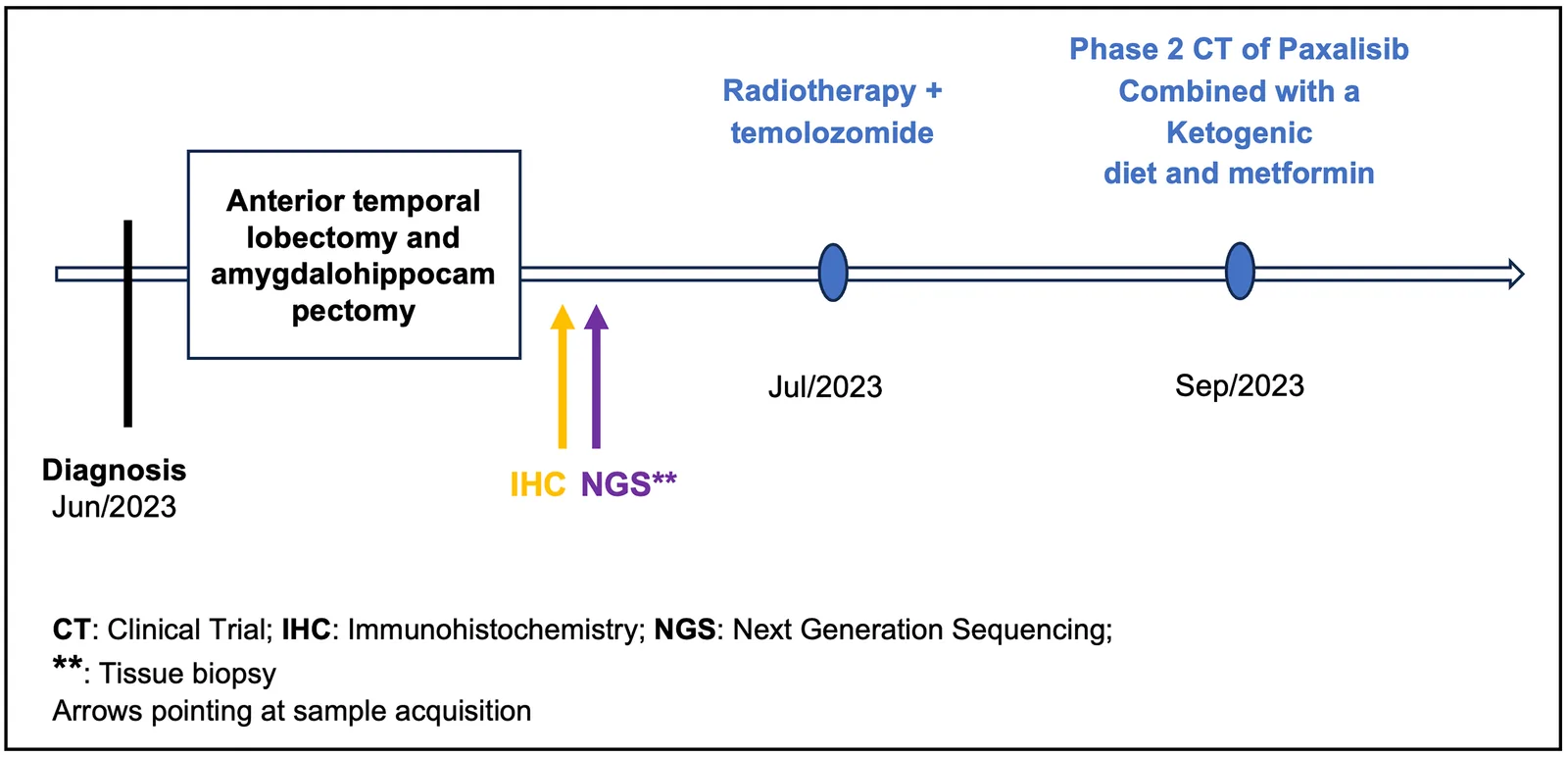
Treatments and molecular tests timeline. The figure outlines the treatments and molecular tests the patient has undergone throughout his medical history.

Histological analysis of the brain tissue revealed high-grade diffuse astrocytoma with epithelial component, which was histologically consistent with GBM.

The patient underwent multiple molecular profiling assessments, as outlined in Table 1. The pathology report performed by the treating institution included immunohistochemistry (IHC) results that were positive for ATRX, GFAP, CD34 and synaptophysin. No evidence of *BRAF-V600E* and *H3-K27M* mutations were found. The methylation profile indicated an *IDH*-wild type tumor, matching the GBM diagnosis [4], and consistent with the retained ATRX positive finding. Also, the tissue was unmethylated for *MGMT* promoter. Results of fluorescence *in situ* hybridization (FISH) test revealed *EGFR* amplification and heterozygous deletion of *CDKN2A*. Since *CDKN2A* and *MTAP* are located adjacent to each other on chromosome 9p21 and are frequently co-deleted, *MTAP* loss could not be excluded based on the available molecular findings [34]; however, *MTAP* expression was not assessed by IHC in this case.

**Table 1:** Molecular alterations

Date of testing		June/2023	June/2023	June/2023	June/2023
**Sample**		Tissue	Tissue	Tissue	Tissue + Blood
**Laboratory**		On-Site	Oncomine Comprehensive	BostonGene	New York Genome Center
**TMB (mutations/mb)**				1.57 (low)	1.23 (low)
**Microsatellite Status**				Stable	Unstable
**Mutations (MAF)**	CDKN2A c.114delC,p.Asn39fs		+ Frameshift deletion 53.8%		+ Frameshift deletion 49%
	EGFR		N/A		
	KMT2C c.5053G>T,p.Ala1685Ser				+ Missense mutation 10%
**Amplifications (CN)**	EGFR	+	+ 116.28	+ >6	
	MDM2		+ 73.61	+ >6	
	PTCH1		+ 7.4		
	HMGA2			+ >6	
**IHC tests**	ATRX	+			
	GFAP	+			
	CD34 and synaptophysin	+			
	BRAF-V600E	−			
	H3-K27M	−			
**Other findings**	MGMT Promoter Methylation	Unmethylated		Unmethylated	
	Methylation Profile	IDH-WT			
	Heterozygous deletion	CDKN2A			
	Tumor microenvironment			• Medium PDL1. • High CD4 and CD8 T-cells. • High T-regs. • Low ECM gene signature.	

Data is provided where available. Only pathogenic alterations are included, and variants of unknown significance (VUS) are not included. Abbreviations: CN: copy number; ECM: extracellular matrix; IHC: immunohistochemistry; TMB: tumor mutations burden; VAF: variant allele frequency; WT: wild-type.

A formalin-fixed paraffin-embedded (FFPE) tissue sample was tested by the Oncomine Comprehensive Next-Generation Sequencing (NGS) assay (80% neoplastic content), confirming *EGFR* amplification. New findings from the Oncomine test included *CDKN2A* frameshift deletion and amplification of *MDM2* and *PTCH1*.

A fresh frozen brain tissue sample was also analyzed by BostonGene (57% neoplastic content), showing a low tumor mutational burden (TMB) and microsatellite stability (MSS). Consistent with previous results, the BostonGene test confirmed unmethylated *MGMT* promoter, *EGFR* amplification, *CDKN2A* loss, and *MDM2* amplification. New discoveries included *EGFR* loss of heterozygosity and *HMGA2* amplification. The tumor microenvironment (TME) showed moderate PD-L1 expression and significant immune infiltration with a high number of CD4 T-cells, CD8 T-cells and T-regs, while angiogenesis and extracellular matrix gene signatures were low.

Additionally, the New York Genome Center conducted another molecular profiling assessment, which involved whole-genome sequencing of DNA from FFPE brain tissue and matched blood, as well as RNA transcriptome sequencing of FFPE brain tissue. The TMB was low, consistent with previous tests, but the assessment indicated microsatellite instability (MSI), in contrast to the MSS result from the BostonGene test. This discordance was not resolved by orthogonal testing, as IHC for mismatch repair proteins or another validated MSI/MMR assay was not performed. The *CDKN2A* frameshift mutation was also detected here, along with a new missense mutation in *KMT2C*. Notably, the variant allele frequency (VAF) for *KMT2C* was 10%, below the assay’s detection limit of 15%, so the mutation was confirmed by RNA sequencing. Additional genomic alterations are listed in Supplementary Tables 1 and 2.

In July 2023, the patient reported a slight decrease in sensation in his right leg compared to the left. At that point, he began chemotherapy treatment with temozolomide (75 mg/m^2^) and radiotherapy to the tumor bed for six weeks, during which no progression was detected. However, given the post-surgical treatment setting, response to temozolomide could not be definitively assessed separately from the effects of surgery and radiotherapy. Although temozolomide is part of standard first-line therapy for GBM, the patient’s unmethylated MGMT promoter status suggested a reduced likelihood of benefit from temozolomide compared with MGMT-methylated disease. In September 2023, the patient enrolled in a phase II clinical trial for newly diagnosed and recurrent GBM, receiving paxalisib (PI3K/mTOR inhibitor) combined with ketogenic diet and metformin. Trial enrollment was pursued as an investigational post-chemoradiotherapy strategy in the context of high-risk MGMT unmethylated GBM and the need for additional therapeutic approaches beyond standard treatment. The rationale for the paxalisib treatment was based on the frequent *PTEN* alterations in brain tumors and the common aberrations in the PI3K pathway in such tumors.

At the time of the MTB discussion, the patient was in remission while undergoing treatment with paxalisib, the ketogenic diet, and metformin, and he was considering a personalized neoantigen vaccine therapy in Germany (https://classic.clinicaltrials.gov/ct2/show/NCT05356312).

## DISCUSSION

The WIN Consortium International MTB discussed potential therapy options in the event of disease progression, acknowledging that relapse is likely to occur at some point. The MTB considered the patient’s comprehensive molecular profiling while also taking into account that the patient is also considering pursuing a vaccine therapy.

One limitation of the molecular interpretation was the discordant microsatellite status reported by two assays, with the BostonGene test indicating MSS and the New York Genome Center assessment indicating MSI. This discordance may reflect differences in assay methodology, tumor sampling, or intratumoral heterogeneity, as GBM can show substantial spatial molecular features [35]. Because MSI/MMR status may have therapeutic implications, particularly when considering immune checkpoint blockade (ICB), this result should be interpreted with caution. Orthogonal confirmation by IHC for mismatch repair proteins or another validated MSI/MMR assay would be required before using MSI status to guide treatment decisions.

The amplification of both *MDM2* and *EGFR* poses challenges for the patient when considering immune checkpoint inhibitor options, as these alterations are associated with resistance and hyperprogression [36]. While *EGFR* is less strongly linked to ICB related hyperprogression than *MDM2*, its alteration is still closely associated with resistance [37].

ERAS-801, an *EGFR* inhibitor with high central nervous system penetration being investigated in the THUNDERBBOLT-1 phase I study for recurrent GBM, recently received FDA fast-tracked status, and could have been a potential option if it was still open for enrollment [38, 39]. Alternatively, any available clinical trials offering *EGFR* inhibitors for this indication could be considered.

*MDM2* amplification has been linked to suppression of the cytotoxic immune response to tumors, with studies showing its role in hyperprogression following ICB [37, 40, 41]. This amplification is also recognized as a poor prognostic factor across multiple cancer types [42], including GBM. *MDM2* is likely a driver in this patient’s tumor and may pose challenges for the immune system’s ability to fight the tumor, including in the context of potential vaccines, which are often given together with anti-PD-1 agents. Residual tumor cells with *MDM2* amplification could evade immune detection and become resistant to immune cell attack. In a pan-cancer analysis of ICB, both *MDM2* and *MDM4* amplifications were associated with poor outcomes in TP53-wildtype breast cancer. However, among ICB-treated non-small cell lung cancer (NSCLC) patients, *MDM4* amplification was associated with reduced survival [42]. A preclinical study demonstrated that an *MDM2* inhibitor (AMG-232) can enhance T-cell mediated killing and provide additional benefits when combined with immune checkpoint inhibitors like pembrolizumab, further boosting T-cell activity [40, 43]. Moreover, *MDM2* and *MDM4* amplification has been observed in brain cancers as well as in metastases to the brain from other primary cancers [44, 45]. In this context, *MDM2* inhibition may represent a biologically relevant therapeutic strategy; however, no *MDM2* inhibitor is currently approved for GBM, and access would generally require enrollment in a clinical trial. Therefore, *MDM2* inhibitors such as navtemadlin, idasanutlin, milademetan, or other agents should be considered investigational in this setting. Clinical trials evaluating MDM2 inhibition in GBM or in molecularly selected MDM2-amplified solid tumors may provide potential route for access, although trial availability, eligibility criteria, CNS activity, and the ability to combine with other matched therapies would need to be assessed at the time of relapse. Examples of clinical trials evaluating MDM2-pathway inhibition in GBM or molecularly selected tumors are summarized in Supplementary Table 3.

Tailored drug combinations could also be considered at the time of relapse to target both *EGFR* amplification and *CDKN2A* loss, which are commonly observed in GBM patients. The presence of *CDKN2A* loss raises the possibility of concurrent *MTAP* loss because of their proximity on chromosome 9p21 [34]. Therefore, orthogonal assessment of *MTAP* expression by IHC could be considered in the future, as confirmation of *MTAP* loss may support consideration of clinical trials evaluating PRMT5 inhibitors or other *MTAP*-directed synthetic-lethality approaches [46–48].

This strategy of personalized combination therapies builds upon the I-PREDICT and WINTHER trials [49–53] and is further supported by reports of exceptional responders in high-grade gliomas, highlighting the promise of biomarker-matched targeted therapy and the need for prospective evaluation [54]. For patients with *EGFR* amplification, therapy could include a small molecule inhibitor, such as osimertinib or lapatinib, combined with an antibody such as cetuximab, as this approach has shown synergistic effect both preclinically and clinically [55, 56]. Although the use of antibodies and some small molecule targeted therapies in GBM may be questioned due to concerns about their ability to cross the blood-brain barrier, many GBM patients already have a disrupted barrier [57, 58], which may actually facilitate intratumoral exposure to both antibodies and targeted agents with otherwise limited central nervous system penetration. The combination of osimertinb or lapatinib with cetuximab should be paired with abemaciclib (*CDK4* and *CDK6* inhibitor), which is known to cross the blood-brain barrier [59]. The rationale for this combination lies in the fact that *CDKN2A* loss results in dysregulation of the p16-CDK4/6-RB pathway and increased *CDK4/6* activity [60, 61]. When considering this novel combination, initial doses of the three agents should be reduced by about half to ensure the patient’s tolerance. In addition, the patient should initially be monitored weekly through clinic visits and laboratory tests, including complete blood count with differential, platelet count and metabolic profile, to assess toxicity and determine whether dose adjustments are necessary.

A theoretical combination involving an *MDM2* inhibitor, an *EGFR* inhibitor, and ICB was also discussed, primarily in reference *to PD-1*/*PD-L1* inhibition rather than *CTLA-4* blockade or other forms of immunotherapy. Because *MDM2* inhibitors are investigational and are not currently approved for GBM, such a strategy would only be feasible if an *MDM2* inhibitor were available through a clinical trial that permitted combination therapy, through expanded access, or through another appropriate regulatory pathway. This combination raises an important question: should *PD-1*/*PD-L1* blockade be administered simultaneously with both *EGFR* and *MDM2* inhibitors, and could this approach possibly overcome the resistance or accelerated progression that can occur with ICB in the presence of *EGFR* or *MDM2* alterations? *MDM2* is known to inhibit, or at least reduce, T-cell-mediated tumor cell killing, independent of ICB [40, 41]. Thus, combining *MDM2* inhibitor with immunotherapy could also enhance T-cell activity in tumors with *MDM2* amplifications. Notably, hyperprogression related to *MDM2* and *EGFR* alterations has been specifically observed with anti-*PD-1* agents as immunotherapy [37]. This raises the question of whether resistance and hyperprogression associated with *MDM2* and *EGFR* alterations, which have been mainly described in the context of *PD-1*/*PD-L1* ICB, would also extend to *CTLA-4* or other immunotherapeutic approaches. The mechanism responsible for immunotherapy related resistance or hyperprogression in the presence of *EGFR* or *MDM2* alterations remains unknown.

An alternative treatment option, teserpaturev (Delytact), which is currently only available in Japan, may be considered if the patient was willing to travel. This is a genetically engineered herpes simplex virus type I (strain F) in which the α47 gene and both copies of the γ34.5 gene have been deleted, and the infected cell protein 6 (ICP6) gene has been inactivated by the insertion of the lacZ gene from Escherichia coli. Teserpaturev injection, when intratumorally administered to patients with malignant glioma, is expected to produce two main effects: (1) the mutant virus selectively replicates in tumor cells, causing destruction of the infected cells through the replication process, exerting a cytocidal effect, and (2) the treatment stimulates the induction of tumor-responsive T cells, which activates antitumor immunity and thus prolongs the survival of patients with malignant glioma [62]. The BostonGene TME profile may provide additional context for this strategy. The report suggested a T-cell-infiltrated TME, with increased CD4+ and CD8+ T-cell infiltration, intermediate PD-L1 expression, and relatively low angiogenesis and extracellular matrix signature, suggesting a microenvironment that may be relatively permissive to immune-cell infiltration. These features could support the biological rationale of an immune-activating local therapy such as teserpaturev, whose proposed mechanism includes induction of tumor-responsive T cells. However, the concomitant abundance of regulatory T cells suggests a counter-regulated immunosuppressive component that could limit effective antitumor immunity. Therefore, the TME profile provides biologic context supporting cautious consideration of immune-modulating approaches, but should not be interpreted as sufficient evidence to establish expected clinical benefit from teserpaturev. There was also discussion about using circulating tumor DNA (ctDNA) to monitor the patient’s recurrence. However, some studies suggest that blood-derived ctDNA in GMB patients almost entirely reflects clonal hematopoiesis of indeterminate potential (CHIP) rather than tumor derived DNA) [63]. These findings underscore the challenges associated with using ctDNA to predict relapse in brain tumor patients.

Overall, the MTB prioritized clinical trial enrollment at relapse, particularly trials evaluating *EGFR*-targeted therapy, *MDM2*-pathway inhibition, immune-modulating strategies, or rational biomarker-matched combinations. In the absence of a suitable trial, the leading personalized therapeutic considerations included *EGFR*-directed therapy combined with *CDK4/6* inhibition to address *EGFR* amplification and *CDKN2A* loss, while *MDM2*-directed approaches remained investigational and dependent on clinical trial or other regulated access. This case highlights how international MTBs can integrate clinical context, genomic alterations, and TME features to identify novel matched therapeutic options for patients with GBM.

## SUPPLEMENTARY MATERIALS


