# Supplementary material for: Case Report WIN-MTB-2024001 – WIN International Molecular Tumor Board: A 55-year-old male with glioblastoma exhibiting amplifications in MDM2 and EGFR
Source: Oncotarget. 2026 Oct 16;17:462–75. doi: 10.18632/oncotarget.28920 (PMC13718356; doi:10.18632/oncotarget.28920)
Supplement: Supplementary file 1 [file oncotarget-17-28920-s001.pdf]

Case Report WIN-MTB-2024001 – WIN International Molecular Tumor Board: A 55-year-old male with glioblastoma exhibiting amplifications in MDM2 and EGFR

SUPPLEMENTARY MATERIALS

Supplementary Table 1: Genomic alterations detected by New York Genome Center

Chromosome	Start base position	End base position	Size (bp)	Genes	Type
4p16.3q12	68,596	52,936,603	52,868,008	RHOH, PHOX2B, FGFR3, SLC34A2, TEC, WHSC1, N4BP2	Loss
4q12	52,936,604	57,122,400	4,185,797	PDGFRA, FIP1L1, CHIC2, KDR, KIT	Gain
4q12q22.1	57,122,401	88,601,696	31,479,296	PTPN13, AFF1	Loss
4q22.1q28.3	91,280,241	136,837,039	45,556,799	IL2, RAP1GDS1, TET2, FAT4, LEF1	Loss
4q31.23q31.3	150,984,070	153,524,445	2,540,376	FBXW7	Loss
4q32.1q35.2	158,853,972	191,044,164	32,190,193	CASP3, DUX4L1, FAT1	Loss
6p25.3p25.1	147,786	6,591,695	6,443,910	IRF4	Loss
6p11.1q27	58,779,884	171,049,448	112,269,565	SGK1, PRDM1, GOPC, RSPO3, BCLAF1, TNFAIP3, EZR, FGFR1OP, EPHA7, CCNC, FOXO3, ROS1, PTPRK, MYB, ECT2L, ESR1, QKI, MLLT4, ARID1B	Loss
7p22.3q36.3	10,005	159,128,563	159,118,559	HNRNPA2B1, HOXA9, HOXA11, HOXA13, SFRP4, CARD11, PMS2, RAC1, ETV1, JAZF1, FKBP9, IKZF1, EGFR, FAM131B, MNX1, ZNF479, ELN, GRM3, EZH2, SBDS, HIP1, AKAP9, POT1, SND1, SMO, CREB3L2, TRIM24, KIAA1549, TRRAP, CUX1, MET, BRAF, CNTNAP2, KMT2C, CDK6	Gain
7p11.2	54,768,637	55,519,865	751,229	EGFR	Gain
8p23.3p12	31,278	31,984,310	31,953,003	LEPROTL1, ARHGEF10, WRN, PCM1	Loss
8p12q24.3	35,266,842	146,303,880	111,037,039	WHSC1L1, IKBKB, FGFR1, ANK1, HOOK3, PLAG1, CHCHD7, HEY1, COX6C, MYC, RECQL4, TCEA1, RUNX1T1, PABPC1, EIF3E, RAD21, NDRG1, PREX2, NCOA2, NBN, CDH17, UBR5, EXT1, FAM135B, CNBD1, CSMD3, RSPO2	Loss
9p24.3p21.1	10,000	32,847,640	32,837,641	CD274, PDCD1LG2, PSIP1, CDKN2A, JAK2, NFIB, MLLT3, PTPRD	Loss
9p13.3q13	33,584,061	66,454,673	32,870,613	FANCG, PAX5	Loss
9q21.2	79,308,312	80,414,238	1,105,927	GANQ	Gain
10p15.3q26.3	63,446	135,242,577	135,179,132	KLF6, NCOA4, PRF1, NUTM2B, NUTM2A, NFKB2, GATA3, KIF5B, RET, A1CF, BMPR1A, FAS, CYP2C8, LARP4B, ABI1, ZEB1, CCDC6, TET1, KAT6B, PTEN, CPEB3, TLX1, SUFU, TCF7L2, KIAA1598, FGFR2, MGMT, MLLT10, NT5C2, VTI1A	Loss
11q13.1	64,752,262	65,479,660	727,399	MALAT1	Gain
12q14.2q14.3	64,868,628	67,656,829	2,788,202	WIF1, HMGA2	Gain
12q15	68,769,939	69,753,356	983,418	MDM2	Gain
12q15	70,736,230	71,019,838	283,609	PTPRB	Gain
14q11.2q13.1	20,424,466	34,946,863	14,522,398	CCNB1IP1, ARHGAP5	Loss
14q21.1q21.3	38,036,685	49,817,055	11,780,371	FOXA1	Loss
14q23.1q24.2	58,157,154	73,053,178	14,896,025	SIX1, HIF1A, MAX, RAD51B, GPHN	Loss
15q11.1q26.3	20,000,005	102,521,290	82,521,286	NUTM1, FES, KNSTRN, CASC5, B2M, USP8, POLG, IDH2, BUB1B, MYO5A, TCF12, MAP2K1, SMAD3, PML, NTRK3, CRTC3, BLM, CHD2	Loss
22q13.1	38,776,969	40,523,778	1,746,810	APOBEC3B, PDGFB	Loss

Supplementary Table 2: Somatic variants of uncertain clinical significance detected by New York Genome Center

Gene	Ensemble ID	Chr :Position	Variant	Type	VAF
ATG2B	ENST00000359933	chr14:96777947	c.3922C>T,pArg1308Cys	NON_SYNONYMOUS_CODING	39%
C9orf171	ENST00000343036	chr9:135374822	c.467A>T,p.Tyr156Phe	NON_SYNONYMOUS_CODING	57%
CADPS2	ENST00000449022	chr7:122303397	c.679delA,p.Ser227fs	FRAME_SHIFT	21%
CASKIN2	ENST00000321617	chr17:73500047	c.1468-4G>A,NA	SPLICE_SITE_REGOIN+INTRON	43%
CNNM1	ENST00000356713	chr10:101090267	c.1123C>A,p.Pro375Thr	NON_SYNONYMOUS_CODING	16%
CTD-2162K18.4	ENST00000590750	chr19:37266387	c.233T>C,p.Val78AIa	NON_SYNONYMOUS_CODING	50%
CYP2A7	ENST00000301146	chr19:41386153	c.494-4G>A,NA	SPLICE_SITE_REGOIN+INTRON	45%
DKKL1	ENST00000221498	chr19:49878137	c.581G>A,p.Arg194Gin	NON_SYNONYMOUS_CODING	31%
FAT3	ENST00000298047	chr11:92531590	c.5411A>T,p.Asp1804Val	NON_SYNONYMOUS_CODING	51%
GRIA2	ENST00000323661	chr4:158142482	c.-266A>T,NA	START_GAINED+UTR_5_PRIME	32%
HEPH	ENST00000519389	chrX:65478715	c.3092G>T,p.Gly1031Val	NON_SYNONYMOUS_CODING	18%
HSPG2	ENST00000374695	chr1:22155546	c.12019C>T,p.Pro4007Ser	NON_SYNONYMOUS_CODING	47%
JMJD4	ENST00000366758	chr1:227921714	c. 586G>A,p.Val196lle	NON_SYNONYMOUS_CODING	49%
KIAA0947	ENST00000296564	chr5:5464339	c.4892C>G,p.Pro1631Arg	NON_SYNONYMOUS_CODING	29%
MAST4	ENST00000404260	chr5:66430465	c.2350G>A,p.Val784Met	NON_SYNONYMOUS_CODING	19%
MROH2A	ENST00000389758	chr2:234737654	c. 4382G>A,p.Arg1461Gln	NON_SYNONYMOUS_CODING	45%
MS4A6E	ENST00000300182	chr11:60105244	c.178A>G,p.Ser60Gly	NON_SYNONYMOUS_CODING	54%
NLRP3	ENST00000336119	chr1:247588075	c.1330G>A,p.Val444lle	NON_SYNONYMOUS CODING	36%
NRF1	ENST00000311967	chr7:129330253	c.473A>G,p.Lys158Arg	NON_SYNONYMOUS_CODING	21%
OR2G3	ENST00000320002	chr1:247769291	c.404T>C.p.lle135Thr	NON_SYNONYMOUS_CODING	49%
OR5P2	ENST00000329434	chr11:7817666	c.824C>T,p.Thr275lle	NON_SYNONYMOUS CODING	50%
OR7G2	ENST00000305456	chr19:9213600	c.383T>C,p.Phe128Ser	NON_SYNONYMOUS_CODING	44%
PCDH20	ENST00000409186	chr13:61986988	c.1244C>A.p Ser415Tyr	NON_SYNONYMOUS_CODING	50%
PDZD8	ENST00000334464	chr10:119044515	c.1729C>G.p.Gln577Glu	NON_SYNONYMOUS_CODING	18%
PKHD1	ENST00000371117	chr6:51914979	c.2255C>T,p.Thr752Met	NON_SYNONYMOUS_CODING	54%
QKI	ENST00000361752	chr6:163983094	c.628_634dupAAATCAC,NA	SPLICE_SITE_DONOR+INTRON	44%
RAB39A	ENST00000320578	chr11:107799343	c.49T>A.p.Ser17Thr	NON_SYNONYMOUS_CODING	38%
RAPGEF2	ENST00000264431	chr4:160252975	c.1286G>C.p.Arg429Pro	NON_SYNONYMOUS_CODING	45%
ROGDI	ENST00000586504	chr16:4847751	c.514C>T,p.Arg172Cys	NON_SYNONYMOUS_CODING	58%
RSAD1	ENST00000258955	chr17:48557021	c.167G>A,p.Cys56Tyr	NON_SYNONYMOUS_ CODING	46%
SAMD4B	ENST00000314471	chr19:39871325	c.1748T>G,p.Leu583Arg	NON_SYNONYMOUS_CODING	16%
SCYL1	ENST00000270176	chr11:65302732	c.1265C>T,p.Ala422Val	NON_SYNONYMOUS_CODING	22%
STX2	ENST00000392373	chr12:131293256	c.281-7T>C,NA	SPLICE_SITE_REGION+INTRON	18%
SUCLA2	ENST00000378654	chr13:48547466	c.596C>A,p.Ser199Tyr	NON_SYNONYMOUS_CODING	52%
SV2B	ENST00000330276	chr15:91795210	c.613C>T,p.Arg205*	STOP_GAINED	76%
TMEM132C	ENST00000435159	chr12:128899621	c.430C>T,p.Arg144 Trp	NON_SYNONYMOUS_CODING	35%
TTN	ENST00000589042	chr2:179428474	c.82385C>T,p.Thr27462Met	NON_SYNONYMOUS CODING	18%
XDH	ENST00000379416	chr2:31606023	c.887-5C>T,NA	SPLICE_SITE_REGION+INTRON	59%

Abbreviation: VAF: variant allele frequency.

Supplementary Table 3: Examples of clinical trials evaluating MDM2-pathway inhibition relevant to GBM or MDM2-amplified tumors

Trial	Agent	Target	Population	Phase	Relevance
NCT03107780	Navtemadlin/ KRT-232/ AMG-232	MDM2 inhibitor	Newly diagnosed or recurrent GBM	Phase 0/I	GBM-specific trial evaluating MDM2 inhibition, including tumor penetration/pharmacodynamic activity
NCT03654716	ALRN-6924/ sulanemadlin	Dual MDM2/ MDMX inhibitor	Pediatric relapsed/refractory solid tumors, brain tumors, lymphoma, or leukemia	Phase I	Includes brain tumors; relevant to MDM2/MDMX pathway inhibition but not adult GBM-specific
NCT05012397	Milademetan	MDM2 inhibitor	Advanced or metastatic solid tumors refractory or intolerant to standard-of-care therapy, with wild-type *TP53*and *MDM2* copy number ≥8 by prespecified biomarker criteria	Phase II basket	Molecularly selected MDM2-amplified tumors; relevant to MDM2 amplification but not GBM-specific
NCT03449381	Brigimadlin/ BI 907828	MDM2 inhibitor	Advanced solid tumors	Phase Ia/Ib	Relevant as an investigational MDM2 inhibitor in solid tumors, but not GBM-specific; trial eligibility, CNS activity, and feasibility for GBM would require case-specific assessment
